# How to distinguish thoracic and cervical lymph nodes during minimally invasive esophagectomy

**DOI:** 10.1111/1759-7714.14554

**Published:** 2022-07-18

**Authors:** Taidui Zeng, Maohui Chen, Bingqiang Cai, Wei Zheng, Chi Xu, Guobing Xu, Chun Chen, Bin Zheng

**Affiliations:** ^1^ Key labortatory of Cardio‐Thoracic Surgery (Fujian Medical university), Fujian Province University Fuzhou China; ^2^ Department of Thoracic Surgery Fujian Medical University Union Hospital Fuzhou China

**Keywords:** esophageal cancer, thoracic and cervical lymph nodes, three‐dimensional reconstruction

## Abstract

**Purpose:**

In this article, we aimed to reconstruct the cervical–thoracic junction plane (CTJP) using a three‐dimensional (3D) reconstruction system. Thus, the CTJP can be judged during surgery to better distinguish cervical–thoracic lymph nodes.

**Methods:**

We included patients in Fujian Medical University Union Hospital from December 2019 to March 2020. All patients underwent a thin‐slice and enhanced computed tomography scan of the chest with 3D reconstruction using the IQQA system (EDDA technology) to reconstruct the CTJP, brachiocephalic trunk, right common carotid artery, and right subclavian artery. The distance from the intersection of the right subclavian artery and the CTJP to the origin of the right subclavian artery (ORSA) was measured, and the relationship between this distance and the patient's sex, BMI and height was analyzed.

**Results:**

Seventy‐three patients were enrolled, of whom 12 had ORSA above the CTJP, while 61 had ORSA below the plane. There was a significant difference in age between the two groups (*p* = 0.04), compared with height, weight and BMI (*p* > 0.05). In 61 patients with the ORSA below the CTJP, the average distance was 24.7 ± 7.6 mm. The difference between the distance and BMI (*p* = 0.02) was statistically significant, and it was increased with increasing BMI.

**Conclusions:**

The relationship between the ORSA and CTJP can be clarified through 3D reconstruction. The cervical‐thoracic recurrent laryngeal nerve lymph nodes can be distinguished clearly in minimally invasive esophagectomy, contributing to the accurate N staging of middle‐thoracic esophageal cancer.

## Graphical abstract

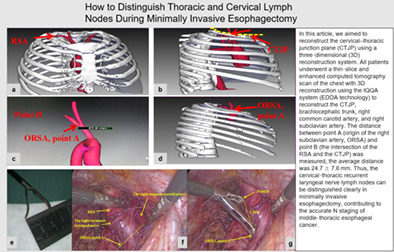

## BACKGROUND

Esophageal cancer is the eighth most common cancer worldwide. The incidence of esophageal cancer in China ranks first in the world. The incidence rates of men and women rank third and fifth, respectively, and the mortality rate is fourth.^1^, ^2^ The location and number of lymph node metastasis is one of the important factors affecting the prognosis of patients with esophageal cancer.^3^, ^4^ In recent years, with the development of minimally invasive surgery for esophageal cancer, adjuvant treatment of esophageal cancer, and other new technologies, surgeons have gradually reached consensus on standardized treatment of esophageal cancer.^5^, ^6^ The treatment of esophageal cancer is comprehensive and based on surgery, with surgical resection the most effective treatment in the early and middle stages.^7^, ^8^, ^9^, ^10^ In recent years, with the development of endoscopic instruments and surgical techniques, the application of thoracoscopy in minimally invasive esophagectomy (MIE) has attracted much attention in the field of international esophageal surgery. It has been proven that compared with open surgery, MIE can shorten the length of hospital stay and reduce the rate of postoperative hoarseness, pulmonary complications, and other complications.^11^, ^12^, ^13^ Moreover, thoracoscopic resection of esophageal cancer is safe and feasible in the removal of recurrent laryngeal nerve (RLN) lymph nodes and can achieve radical mediastinal lymph node dissection.^14^ Owing to the high frequency of RLN lymph node metastasis in patients with esophageal squamous cell carcinoma (ESCC),^15^, ^16^, ^17^ mediastinal lymphadenectomy is recommended for accurate staging and possible tumor clearance.^18^ However, bilateral paralaryngeal nerve lymph nodes are located in the bilateral tracheoesophageal sulcus of the upper mediastinal stenosis, which is the highest position in the chest and the most difficult to clean.^19^ In recent years, our department has adopted methods such as the semi‐prone‐position, low‐pressure CO_2_ pneumothorax, single lumen intubation, and suspension of the esophagus to perform thoracic surgery and left RLN lymph node dissection.^20^, ^21^ The operation space is large, the upper mediastinum of patients is well exposed, lymph node dissection under thoracoscopy can be extended to the level of bilateral inferior thyroid arteries, and lymph nodes adjacent to the cervical RLN can be cleaned under thoracoscopy.

Based on the experience of systemic three‐field lymph node dissection for esophageal cancer in the past 30 years, it is generally believed that lymph node metastasis of thoracic esophageal cancer is more likely to occur in the cervical‐thoracic junction and the thoracic–abdominal junction,^18^ so the lymph nodes adjacent to the RLN in the cervical–thoracic junction plane (CTJP) are areas that must be involved in radical resection of esophageal cancer. The CTJP refers to the bordering area between the neck and the chest. The front boundary of this area is the sternum stem, the back boundary is the first thoracic vertebral body, and the boundary on both sides is the first ribs.^22^ Clinically, the first rib, the seventh cervical vertebra, the clavicle, and the suprasternal notch are often used as the cervical–thoracic anatomy boundary.^23^ However, there is no obvious anatomical sign in the operative field of vision at the cervical–thoracic junction, so it is difficult to define the cervical and thoracic RLN lymph nodes during surgery.

At present, the commonly used standards for grouping lymph nodes in esophageal cancer are the American Joint Committee on Cancer (AJCC) and Union for International Cancer Control (UICC) standards^24^ and the Japan Esophageal Society (JES) standards.^25^, ^26^ In the 8th edition of the TNM staging of esophageal cancer published in 2017, the N staging of esophageal cancer was staged according to the number of regional lymph node metastases, but from the perspective of anatomical characteristics, a more reasonable N staging is based on the location of lymph node metastasis. Currently, the more mature N staging system is the JES standard. In this staging system, cervical (group 101) and thoracic RLN lymph nodes (106recR) belong to different regional lymph nodes. For middle esophageal cancer, the right thoracic RLN lymph nodes (106recR) belong to the N1 category, while the cervical RLN lymph nodes belong to the N2 category. Therefore, how to distinguish the 101 and 106 groups of lymph nodes is helpful for the accurate N staging of middle thoracic ESCC. However, there is no report on the identification of the CTJP under thoracoscopy.

With the continuous development of digital medicine, artificial intelligence three‐dimensional (3D) reconstruction technology has been widely used in clinics, achieving visualization of internal anatomical structures of human organs, digitization of disease diagnosis and treatment, and precision of surgical operations. 3D reconstruction technology is based on two‐dimensional (2D) images and integrates and reconstructs 3D images.^27^ It can reconstruct the 3D anatomical structure of the human body based on computed tomography (CT), magnetic resonance imaging (MRI), and other imaging data to fully understand the nature of the lesion and the 3D structure relationship of the surrounding tissues. According to reports, IQQA 3D reconstruction technology (EDDA Technology, Princeton, NJ, USA)^28^ uses 3D visualization technology to reconstruct complex anatomical structures during precise segmentectomy, segmental liver resection, and partial nephrectomy, thereby providing an important reference for formulating surgical plans.^29^, ^30^, ^31^ Our department has introduced a 3D reconstruction surgery planning system (IQQA‐CHEST), which can be used clinically to reconstruct the 3D anatomical structure of the chest. As an important branch of visualization in scientific computing, the basic principle of 3D visualization of medical volume data is to interpolate these discrete data through algorithms on a computer, turn this data into images with an intuitive 3D effect, and use the characteristics of the human visual system to display the 3D shape of object organs to provide some anatomical structure information that cannot be obtained by traditional means and provide visual interaction means for further simulation operation. The 2D image source is used as the original data, also known as the data layer. Then a mapping relationship from data to graph is implemented. Finally, the mapping relationship from data to graph is materialized by a software algorithm so that people can see its rendering results and control the display attributes of the image by using the attribute object function. Thus, the displayed image is more realistic and the 3D visualization effect is improved. Therefore, in this study, the IQQA‐CHEST reconstruction system was used for 3D reconstruction of the CTJP, brachiocephalic trunk, right common carotid artery, and right subclavian artery (RSA). The distance from the intersection of the RSA and the CTJP to the origin of the right subclavian artery (ORSA) was measured, and we determined the relationship between the ORSA (which can be found easily during the surgery) and CTJP through the 3D reconstruction. According to this relationship, the position of CTJP can be inferred from the position of the ORSA during esophagectomy. We can then accurately judge the group 101 and 106 lymph nodes during surgery.

## MATERIALS AND METHODS

This study was a retrospective study. We retrospectively collected consecutive patients who underwent esophageal cancer resection in our department from December 2019 to March 2020. The study was approved by the ethics committee of our hospital and all patients provided informed consent.

Inclusion criteria:

1. Aged 18–75 years.
2. The diagnosis of middle and lower esophageal thoracic cancer was confirmed by gastroscopy, and no metastases in the upper mediastinum and cervical lymph nodes were found.
3. Not receiving neoadjuvant therapy for esophageal cancer.
4. All patients were examined on the same CT machine (GE Revolutionary CT). The patient's clinical data were complete, and all 3D reconstruction images and measurements were performed by the same operator.

Exclusion criteria:

1. History of previous neck and chest surgery.
2. Combined with spinal tumor, scoliosis, thoracic deformity, and rib fracture.
3. Combined with neck and chest mass or enlarged lymph nodes and vascular malformations.

Position for CT examination: the patient is in the supine position, the central line of the body coinciding with the central line of the bed, and the central line positioning lamp on the central line of the body. Patient should raise his (or her) arms to the sides of head. During the scan, the patient needs to breathe deeply and then hold his (or her) breath.

All patients' thin‐slice enhanced CT data were uploaded to be reconstructed using the IQQA 3D reconstruction system. Baseline data and the distance from the intersection of the RSA and the CTJP to the ORSA were analyzed and summarized.

**FIGURE 1:**
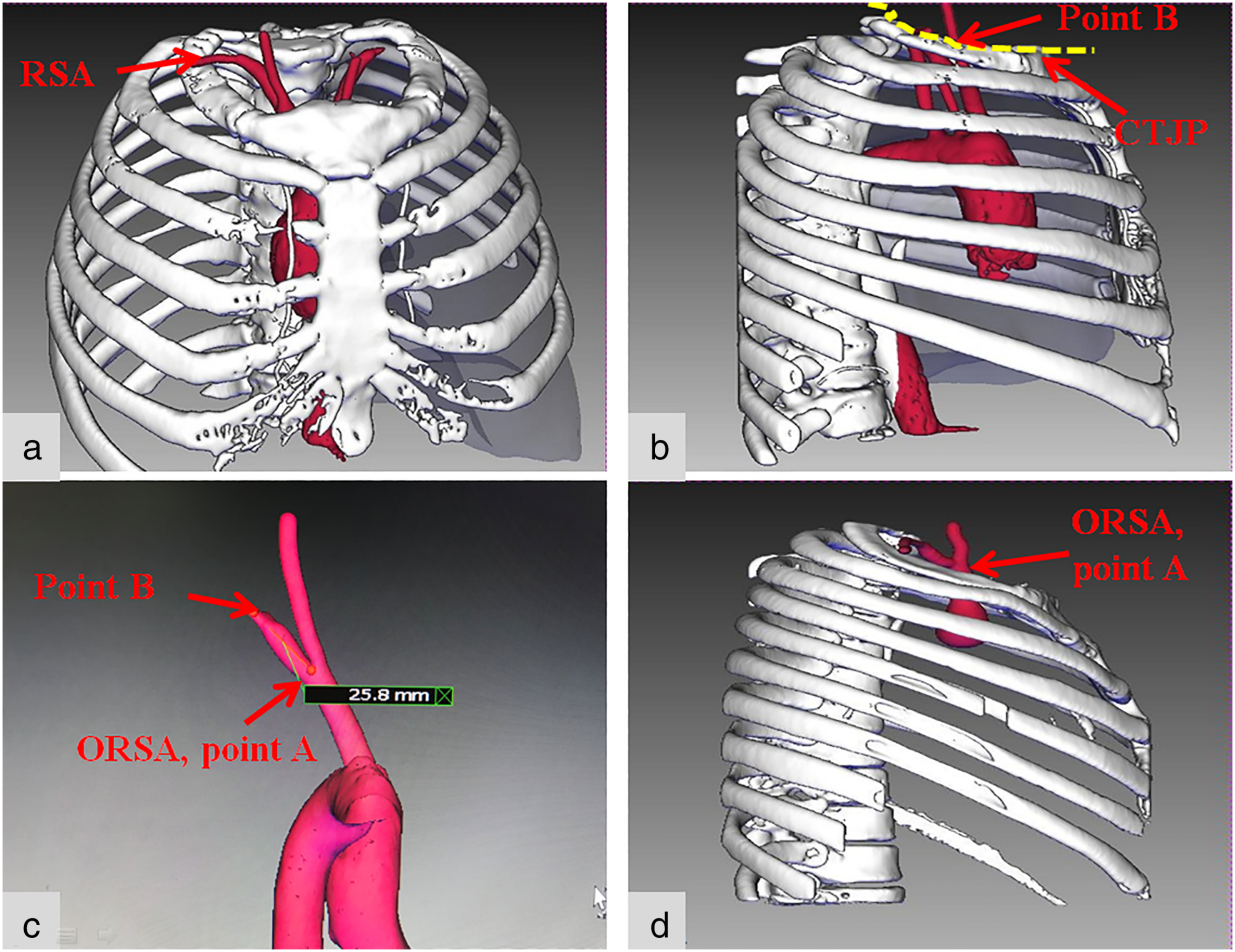
The reconstruction effect. (a–c) The ORSA located below the CTJP. (a) The brachiocephalic trunk, right common carotid artery, RSA. (b) The CTJP and the intersection of the RSA and the CTJP (point B). (c) The distance between point A (ORSA) and point B (the intersection of the RSA and the CTJP) was measured (as shown in this figure, the distance for this patient is 25.8 mm). (d) The ORSA located above the CTJP. RSA, right subclavian artery; ORSA, origin of the right subclavian artery; CTJP, cervical–thoracic junction plane

Reconstruction steps: Thin‐slice enhanced CT of the chest was performed with a thickness of 0.625 mm (maximum 1 mm). Data were uploaded into the 3D reconstruction system, and the aortic arch, brachiocephalic artery, right common carotid artery, RSA, ribcage, and other structures were reconstructed (Figure 1). The CTJP was simulated and the intersection of the RSA and the CTJP was marked to measure the distance from the intersection (point B) to the ORSA (point A).

### Statistical methods

All data were analyzed using SPSS 21.0 statistical software. Measurement data are expressed as mean ± standard deviation. The independent samples *t*‐test was used to compare the two groups. Count data are expressed as the number of cases or *n* (%). The chi‐squared test or Fisher's exact probability method was used to compare the two groups. Statistical significance was set at *p* < 0.05 (two sided).

## RESULTS

The reconstruction effect is shown in Figure 1. A total of 73 patients were enrolled, of which 61 cases (83.6%) demonstrated an ORSA below the CTJP (the below‐plane group, BPG) (Figure 1a–c), while 12 cases (16.4%) demonstrated an ORSA above the CTJP (the above‐plane group, APG) (Figure 1d). The clinical characteristics of the two groups are shown in Table 1. There was a significant difference in age between the two groups (*p* = 0.04), while there was no significant difference in sex, body mass index (BMI), height, or weight between the two groups (*p* > 0.05).

**TABLE 1:** The clinical baseline characteristics of the APG and BPG

Clinical information	Overall (average)	Group A	Group B	*p* value
Patient	73	12 (16.4%)	61 (83.6%)	
Sex				
Female	30 (41.1%)	4 (33.3%)	26 (42.6%)	0.7
Male	43 (58.9%)	8 (66.7%)	35 (57.4%)	
Age (years)	53.8 ± 12.0	59.6 ± 14.4	52.6 ± 11.3	0.04
BMI (kg/m^2^)	23.0 ± 2.7	23.4 ± 3.4	22.9 ± 2.6	0.56
Height (cm)	162.8 ± 6.5	162.8 ± 7.4	162.8 ± 6.4	0.9
Weight (kg)	61.0 ± 8.7	62.0 ± 8.9	60.8 ± 8.7	0.7

Abbreviations: APG, above‐plane group; BMI, body mass index; BPG, below‐plane group.

There were 61 patients in BPG, and the relationships between sex, BMI, weight, height, age, and distance from the ORSA to the CTJP are shown in Table 2. The average height and age in BPG were 162.8 ± 6.4 cm and 52.6 ± 11.3 years, respectively. According to the above height threshold, the patients were divided into a low‐height group (<162.8 cm, *n* = 30) and a high‐height group (≥162.8 cm, *n* = 31), and a low‐age group (<53 years, *n* = 31) and a high‐age group ((≥53 years, *n* = 30). The distance from the ORSA to the CTJP showed no significant difference between the low‐height group and the high‐height group (25.1 ± 5.2 vs. 27.8 ± 9.3 mm, *p* = 0.17) or between the low‐age group and the high‐age group (25.6 ± 6.5 vs. 27.3 ± 8.6 mm, *p* = 0.37).

**TABLE 2:** Relationship between the distance and sex, BMI, weight, height, and age

Clinical information	Patient	Distance	*p* value
Sex	61	26.5 ± 7.6	
Male	26 (42.6%)	28.1 ± 10.0	0.14
Female	35 (57.4%)	25.2 ± 5.1	
BMI			
Normal group (BMI < 24)	46 (75.4%)	25.2 ± 6.5	0.021
Hyper‐recombination group (BMI ≥ 24)	15 (24.6%)	30.4 ± 9.5	
Height (cm)			
Low‐height group (<162.8)	30 (49.2%)	25.1 ± 5.2	0.17
High‐height group (≥162.8)	31 (50.8%)	27.8 ± 9.3	
Age (years)			
Low‐age group (<53)	31 (50.8%)	25.6 ± 6.5	0.37
High‐age group (≥53)	30 (49.2%)	27.3 ± 8.6	

Abbreviation: BMI, body mass index.

According to the BMI (Chinese reference standard),^32^ patients were divided into a normal weight group (BMI < 24, *n* = 46) and a hyper‐recombination group (BMI ≥ 24, *n* = 15). The comparison showed that the distance from the ORSA to the CTJP in the hyper‐recombination group was greater than that in the normal group (25.2 ± 6.5 vs. 30.4 ± 9.5 mm, *p* = 0.021).

**FIGURE 2:**
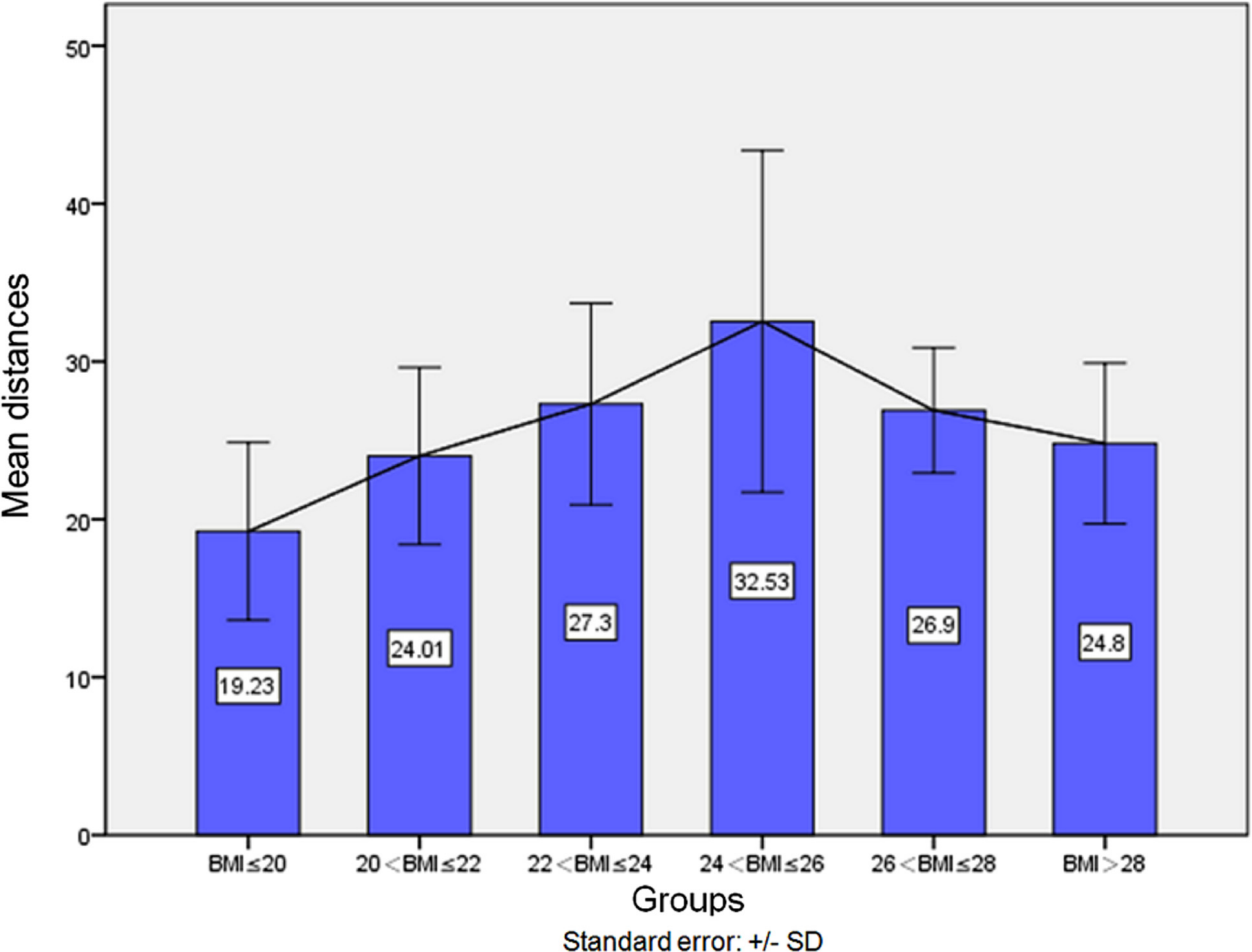
The changing trend in distance between different BMI groups

The relationship between the distance from the ORSA to the CTJP and different BMI groups is shown in Figure 2. According to Figure 2, we found that within a certain range, with the increase in BMI the distance increases, and when 24 < BMI ≤ 26, the distance reaches a maximum. The average distance of this group is 32.5 ± 10.8 mm. When BMI > 26, the distance gradually decreases with increasing BMI.

## DISCUSSION

Lymph node dissection is a key step in radical resection of ESCC.^33^, ^34^ Reliable and clean lymph node dissection is helpful for accurate postoperative pathological staging, ensuring radical resection of the tumor and improving the survival of patients.^35^, ^36^ In the 11th edition of the Japanese Classification of Esophageal Cancer by the Japan Esophageal Society in 2017,^26^ cervical lymph nodes are divided into four groups: group 101 (paraesophageal lymph nodes), group 102 (deep cervical lymph nodes), group 103 (postpharyngeal lymph nodes), and group 104 (supraclavicular lymph nodes). Each component has left and right sides. Usually, the area for esophageal cancer lymph node dissection includes the bilateral cervical lymph nodes (including the cervical paraesophageal lymph nodes and both sides of the clavicle), the upper, middle, and lower mediastinal lymph nodes (including the paraesophageal lymph nodes and RNL lymph nodes), and the upper abdomen lymph nodes (including the lymph nodes around the celiac artery and esophageal hiatus). Postoperative lymph node staging varies with tumor location. For middle esophageal cancer, the right thoracic RLN lymph nodes (106recR) belong to the N1 category, while the cervical RLN lymph nodes belong to the N2 category.

With the continuing popularization of thoracoscopy and the Da Vinci robotic surgery system, combined with the semi‐prone‐position, low pressure CO_2_ pneumothorax, single lumen intubation, suspension of the esophagus, and other methods for thoracic esophageal cancer surgery and left recurrent laryngeal nerve lymph node dissection,^21^ the scope of lymph node dissection under thoracoscopy can be extended to the cervical paraesophageal lymph nodes, therefore the 101 groups of lymph nodes can be dissected on both sides under thoracoscopy. The ability to distinguish the group 101 and group 106 lymph nodes during surgery is helpful to achieve accurate N staging of the middle thoracic ESCC. However, there is no clear anatomical mark to distinguish the two groups of lymph nodes during surgery.^23^ We therefore reconstructed the sternum, the upper edge of the first thoracic vertebra, and the first ribs through 3D reconstruction system. According to the anatomical definition, CTJP was simulated and displayed on the reconstructed image, and the arteries passing through this plane were also reconstructed, including the brachiocephalic trunk, right common carotid artery, and right subclavian artery (which can be found easily during surgery). We determined the relationship between the ORSA (which can be found easily during surgery) and the CTJP through the 3D reconstruction. According to this relationship, the position of the CTJP can be inferred from the position of the ORSA during esophagectomy, and we can then accurately judge the group 101 and 106 lymph nodes during surgery.

In this study, IQQA (EDDA technology) was used for 3D reconstruction of the CTJP, the brachiocephalic trunk, the right common carotid artery, and the RSA. There was a total of 73 reconstructed cases, of which 12 had an ORSA above the CTJP and 61 had an ORSA below the CTJP. By comparing the sex, age, BMI, height and weight of the two groups of patients, we found that the differences in gender, BMI, height, and weight between the two groups were not statistically significant (*p* > 0.05). The average age of APG patients was higher than that of BPG patients, and the difference was statistically significant (59.6 ± 14.4 vs. 52.6 ± 11.3 years, *p* = 0.04). The reasons maybe as the follows: (1) some patients may have vascular anatomical variation, which requires more cases or multicenter studies to support, and (2) elderly patients are more prone to osteoporosis due to low bone mass and reduced bone strength as osteoporosis is a metabolic disease characterized by low bone mass and bone tissue microstructure destruction. According to the literature,^37^, ^38^, ^39^, ^40^ this low bone mass and microstructure destruction manifests earlier and more extensively in the spine than in peripheral bone, which can lead to changes in vertebral body shape, spinal curvature, and mechanical characteristics, of which the changes in the thoracolumbar spine are the most obvious. Deformation of the vertebral body and changes in the curvature of the spine due to old age and osteoporosis may cause changes in the relative positions of the ORSA and CTJP.

According to the lymph node staging standard for ESCC, the right RLN lymph node (group 106recR) is located on the right side of the trachea, distributed along the right RLN, and the lower boundary is located on the lower wall of the RSA. In 12 patients (about 16.4%) the ORSA was located above the CTJP. For these patients, the right RLN lymph nodes were all located above the thoracic entrance, there was no intramediastinal RLN lymph nodes (group 106 recR group), and only the cervical RLN lymph nodes were present (group101). Therefore, for elderly patients 3D reconstruction can be performed before surgery to determine whether the ORSA is above the CTJP in order to perform accurate postoperative lymph node staging.

**FIGURE 3:**
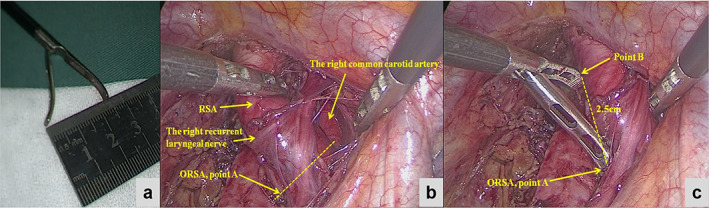
How to judge 2.5 cm during surgery. (a) The distance from the opening of the tail of the gastric forceps during esophageal cancer surgery is approximately 2.5 cm. (b) We dissected the right subclavian artery and the right common carotid artery, then we determined the position of the ORSA. (c) When the lower end was in the ORSA position (point A), the upper end (point B) was in the position of the CTJP

In the BPG, the distance from the intersection of the RSA and the CTJP to the ORSA was measured, and the average distance was 24.7 ± 7.6 mm (approximately 2.5 cm). The ORSA was then used as a landmark to search for the CTJP. However, this distance was still hard to measure during surgery. We measured when the tail of the gastric forceps is open during surgery, the distance between the two tips is approximately 2.5 cm, so we thought that the gastric forceps could be used to assist the judgment of CTJP during surgery (Figure 3). When the lower end was in the ORSA (Figure 1, point A), the upper end was in the intersection of the RSA and the CTJP (Figure 1, point B), which was used as the boundary of the cervical and thoracic lymph nodes to facilitate accurate lymph node staging.

We analyzed the relationship between this distance and sex, BMI, height and age. The results showed that the distance was related to the BMI (*p* = 0.02). We found that when the BMI was ≤26, with increasing BMI, the distance increased. When the BMI was >26, the distance gradually decreased with increasing BMI. We believe that the reasons are as follows: patients with high BMI, and diaphragm and thoracic movement are restricted due to increased adipose tissue, these patients often combined with chest wall hypertrophy and diaphragm elevation. Due to the elevation of the diaphragm, the thorax becomes shorter and the volume of the thorax becomes smaller. Due to the reduction of thoracic volume, the subclavian artery runs smoothly, so the distance increases. At present, the reports on the trend of the subclavian artery is rare, and further clinical studies are needed to support our opinion.

This study has some limitations that should be noted. First, the study design was retrospective, but the patients enrolled in this study strictly met the enrollment criteria, and all patients underwent routine preoperative CT so the risk of selection bias was small. Second, although there is a clear definition of the CTJP in anatomy, we do not have a clear anatomical landmark to distinguish the neck from the chest in the surgery of esophageal cancer. We reconstructed the CTJP as the thoracic entrance. Thus, the definition of the plane of the neck and thoracic junction during surgery requires more research. Third, owing to the small sample size of this study, studies with larger sample sizes need to be conducted to provide more convincing theoretical evidence.

## CONCLUSIONS

The relationship between the ORSA and the CTJP can be clarified through 3D reconstruction. In most patients, the ORSA was located below the CTJP. For these patients, the average distance from the ORSA to the CTJP was 24.7 ± 7.6 mm. With this relationship, the cervical and thoracic RLN lymph nodes can be distinguished in MIE, which contributes to the accurate N staging of middle esophageal cancer.

## CONFLICT OF INTEREST

We declare that there is no conflict of interest.
